# Childhood Sexual Abuse and Indicators of Immune Activity: A Systematic Review

**DOI:** 10.3389/fpsyt.2018.00354

**Published:** 2018-08-06

**Authors:** Ana T. D. D'Elia, Camila T. Matsuzaka, Jair B. B. Neto, Marcelo F. Mello, Mario F. Juruena, Andrea F. Mello

**Affiliations:** ^1^Department of Psychiatry, Federal University of São Paulo, São Paulo, Brazil; ^2^Department of Medicine, Federal University of São Carlos, São Carlos, Brazil; ^3^Department of Psychological Medicine, Institute of Psychiatry, Psychology and Neurosciences, Kings College London, London, United Kingdom; ^4^Department of Neurosciences and Behavioral Sciences, Faculty of Medicine of Ribeirão Preto, University of São Paulo, Ribeirão Preto, Brazil

**Keywords:** childhood sexual abuse, immune activity, inflammatory markers, neurobiology, posttraumatic stress disorder, interleukin-6, cytokines

## Abstract

**Background:** Childhood sexual abuse (CSA) is a prevalent subtype of early life stress associated with changes in immunological and neuroendocrine systems leading to inflammatory responses of the organism and increasing several inflammatory and immune markers. We aimed to conduct a systematic review concerning the association between CSA and indicators of immune activity.

**Methods:** We conducted a search for articles in PubMed, Scopus, PsycINFO, and Web of Science, using the key words: (“Child sexual abuse” OR “childhood maltreatment” OR “sexual violence” OR “posttraumatic stress disorder” OR “rape”) AND (“cytokines” OR “inflammatory markers” OR “interleukin” OR “tumor necrosis factor” OR “C-reactive protein”). PRISMA guidelines were used in order to improve the quality of this research, and MeSH terms were used in PubMed.

**Results:** A total of 3,583 studies were found and, after application of the exclusion criteria, 17 studies were included in this review. Most studies reported an increase of inflammatory activity associated with the presence of early abuse. IL-6, TNF- α, and C-reactive protein were the most frequently analyzed markers and some studies showed higher levels in individuals that suffered CSA compared with controls, although the results were heterogeneous, as was the assessment of CSA, repeated trauma, and time of occurrence. It was not possible to perform a meta-analysis because the results were diversified.

**Conclusion:** CSA is associated with changes in inflammatory markers levels. Improving the assessment of subtypes of trauma is important to further understand the complex correlations of CSA and its biological consequences such as psychiatric and physical illness in later life.

## Introduction

Childhood is a critical period of important brain structure development. Stress and traumatic events during this phase may have persistent consequences in the neurobiological system of children and throughout their lifetime (1). Early life stress is related to emotional and social dysfunction as well as to physical and psychiatric diseases later in life, including diabetes, cardiovascular diseases, cancer, depression, and posttraumatic stress disorder (PTSD) (2–6). Childhood sexual abuse (CSA) is one subtype of early life stress. Conceptualized as inappropriate sexual interaction between an individual who has a relationship of power, trust or responsibility to the minor (7), CSA is a major public health problem that has high prevalence worldwide approximately 1 in 5 girls exposed to CSA (8, 9). CSA is also associated with worse functional abilities, more physical symptoms, greater median annual health care costs, and nearly twice the number of emergency room visits (10).

Childhood sexual abuse is a traumatic event for both children and teenagers that have been implicated in psychological responses to trauma. Many researches have evaluated the biological effects of the trauma on the hypothalamic-pituitary-adrenal (HPA) axis and have demonstrated that childhood maltreatment entails important consequences to the HPA axis functioning which can lead to an imbalance on the stress regulation throughout life (10, 11). The HPA axis is a neuroendocrine system complex component that influences directly on the feedback reactions needed for the acute stress adaptation. This system is also responsible for maintaining the circadian rhythm, and the metabolic and immune functioning (12). Activation of the HPA axis controls the transcription of pro-inflammatory genes and others genes related to antiviral immune response promoting the secretion by adrenal cortex of cortisol, which has a major anti-inflammatory effect on the organism (13). The cortisol regulates an inhibitory effect on the gene transcription mediated by a glucocorticoid receptor which binds to a promoter RNA sequences, interrupting the pro-inflammatory gene activity (14). When an imbalance occurs, a phenomenon called glucocorticoid resistance arises: the immune cells decrease their sensibility to the glucocorticoid anti-inflammatory effects in order to redress for their continuous secretion (15). Due to the glucocorticoid resistance, the HPA axis adaptive processes responsible for the “fight or flight” reactions (adaptable to external stimuli) can cause excessive inflammation, especially if chronically engaged (16).

Immune system function is essential for keeping the body healthy, especially when under threat. The inflammatory responses mediated by pro and anti-inflammatory cytokines pose as a key component to identify, neutralize and eliminate external pathogens. The expression of immune response genes including interleukin-1β (IL-1β), interleukin-6 (IL-6), and tumor necrosis factor-α (TNF-α) regulates the inflammation. The activation of these genes regulates how these pro-inflammatory cytokines responsible for the systemic inflammation are secreted (17). The interaction between the cytokines and the brain is initiated when some brain cells such as microglial cells and astrocytes, that are active on the inflammation, secrete pro-inflammatory cytokines binding themselves to the cytokines receptors present in the brain as a response to the peripheral inflammation (18). These cytokines stimulate the release of dopamine, norepinephrine and serotonin (19), thus initiating a neurochemical cascade that affects directly the behavior. These behaviors have been collectively called sickness behaviors and involve decrease on the appetite, on the daytime activities and on the socialization, which are collectively similar to depressive symptoms (20).

Recent studies have indicated evidences of long-term alterations on the inflammatory response due to childhood maltreatment (21), showing the increase of TNF-α (22), pro-inflammatory cytokines (23) and C-reactive protein (CRP) (24).

There are evidences that different subtypes of trauma generate different consequences on mental health and some subtypes of anxiety could be developed according to the type of maltreatment the child was exposed to (25). Additionally verbal hallucinations in psychosis appear to be associated with sexual abuse (26). A review conducted by Carr et al. compared the impact of different subtypes of childhood traumas in adulthood mental disorders and sexual abuse was correlated to a number of psychiatric pathologies such as mood disorders, psychosis, personality disorders, and psychoactive substance abuse (27). Sexual abuse in adulthood is strongly related to the development of PTSD, almost 45% of women develop PTSD after rape, and there is an increased risk if there is a history of CSA (28, 29).

Other studies corroborates with these results: Chen et al. found a strong correlation between sexual abuse against both men and women, and anxiety, eating and sleeping disorders as well as suicide attempts throughout life (30, 31). Furthermore, childhood sex abuse is a key risk factor for the development of depression (32).

Childhood sexual abuse also affects the endocrine, central nervous, and immune systems; previous reviews and meta-analysis pointed some intriguing results. Baumeister et al. showed in a meta-analysis that the occurrence of specific types of trauma lead to different impacts on the inflammatory markers: physical and sexual abuse presented correlation with a significant increase on TNF-α and IL-6 levels, but not on the CRP ones (33). A recent study (34) reviewed the neurobiological effects on early life trauma including endocrinology, inflammation, genetics, and epigenetics pathways, and alterations in brain imaging by showing studies with different results depending on the trauma sub-type, such as alterations in structural MRI in different cerebral areas comparing sexual abuse to emotional abuse victims (35). The way different traumas interact with inflammation pathways and specific neurobiological consequences of each type of abuse still remains unknown.

The aims of this study are to review the findings from researches focusing on immunological consequences of CSA and understand the correlations between CSA and inflammatory markers.

## Methods

This systematic review was conducted according to Preferred Reporting Items for Systematic Reviews and Meta-Analyses (PRISMA) guidelines (36). The protocol was published on the International Prospective Register of Systematic Reviews website (www.crd.york.ac.uk/PROSPERO) under the registration number CRD42017069437.

We searched for articles indexed on Medline/PubMed, PsycINFO, Scopus, and Web of Science published in English, using the following keywords: (“Child sexual abuse” OR “childhood maltreatment” OR “sexual violence” OR “posttraumatic stress disorder” OR “rape”) AND (“cytokines” OR “inflammatory markers” OR “interleukin” OR “tumor necrosis factor” OR “C-reactive protein”). Medical Subject Heading (MeSH) terms were used in PubMed. Two researchers independently (ATD and CTM) screened and selected studies according to the inclusion and exclusion criteria.

To be included in this review, the articles had to investigate childhood sexual abuse, a specific type of early life stress, so that age of onset of sexual abuse had to be <18 years old. Were included only studies written in English and observational studies analyzing the effects of CSA in both genders, such as case control; cross sectional studies; interventional studies, as clinical trials. Studies that investigated solely other forms of childhood adversity were excluded.

The exclusion criteria were as follows: criticisms, reviews, letters to the editors, responses to clinical cases, studies conducted on animals, essays not related to CSA as an independent variable. Studies displaying patients who suffer from psychotic symptoms or bipolar affective disorder, or any other severe medical conditions and those that evaluated patients who depend on immunomodulatory or anti-inflammatory drugs were also excluded. Furthermore, studies that investigated solely other forms of childhood adversity or did not specify the subtype of CM were not compiled in this review.

**Figure 1:**
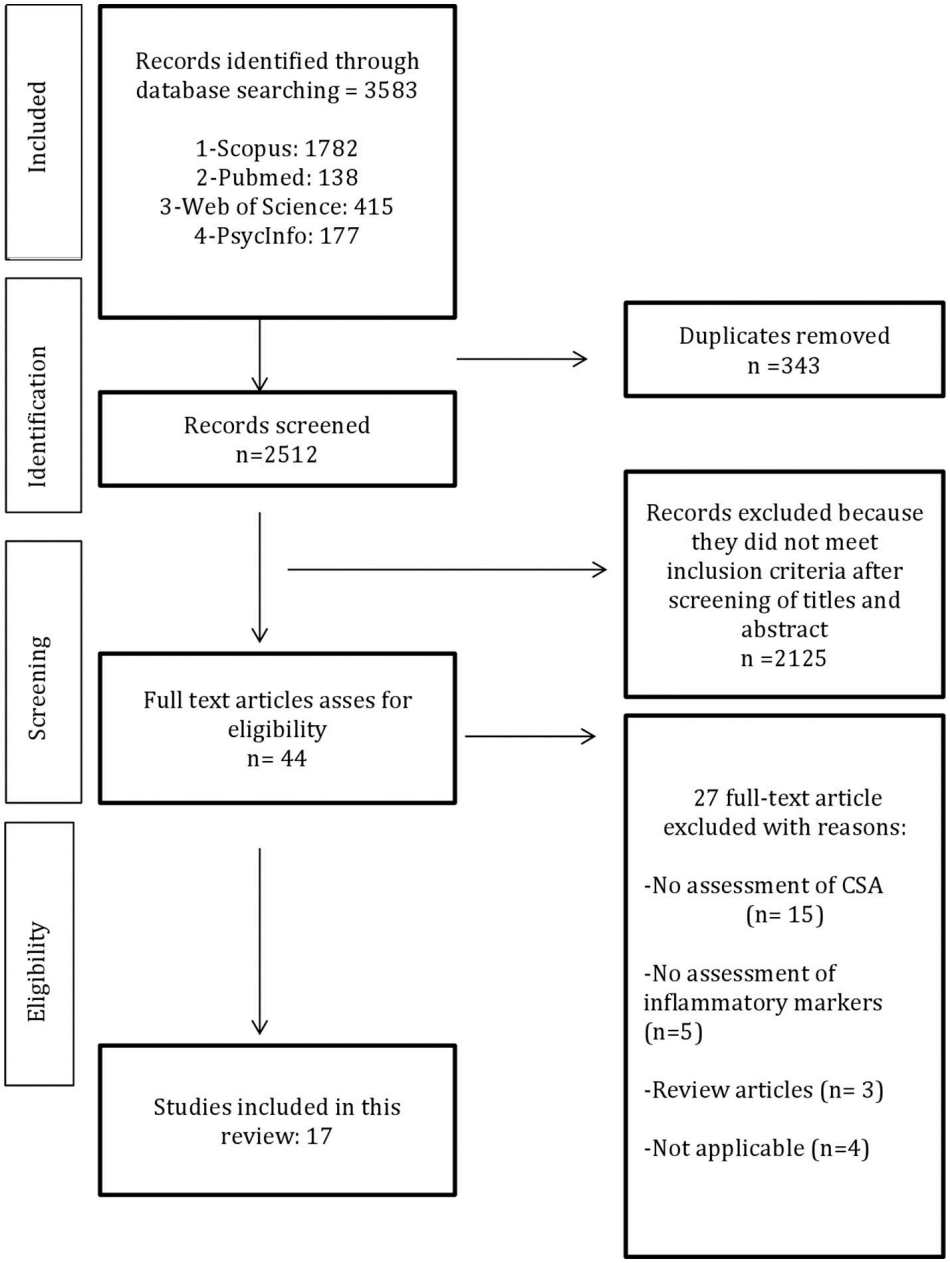
Flow chart of methods.

After removing duplicate studies by hand, the authors selected by title and abstract 44 studies that were read entirely. In order to reach a consensus, the reviewers discussed any possible inconsistencies and in case of doubt a third reviewer (JBN) was asked for an opinion on whether to include or exclude the study in this review. It is important to note that all studies included in this review had to clearly identify the CSA, even if the study evaluated diverse subtypes of maltreatment, the inclusion was only made if the isolated analysis of specific CSA was possible. The methodological scheme showing the criterion for the selection of the articles is shown in Figure 1.

## Results

The research discovered 3,583 papers and after removing duplicated data, 3,196 studies have been excluded.

After screening their titles and abstracts, excluding those papers that did not meet the inclusion criteria, a total of 44 full texts were reviewed independently by two authors. Seventeen studies were included, which analyzed the association between CSA and inflammatory markers. The characteristics extracted from each study were: first author's name, publication year, measured inflammatory markers, age, and number of patients, ethnicity, age of abuse onset, study design, trauma assessment, type of diagnostic instrument used and type of assay. Most of the researches presented a cross-sectional design. The results obtained are summarized in Table 1.

**Table 1:** Characteristics of studies included in this review.

**Articles**	**Inflammatory markers assessed**	**Subjects**	**Ethnicity**	**Abuse age**	**Mean age**	**Design**	**Trauma assessment**	**Other scales**	**Covarietes**	**Assay**	**Main results**
**(****37****) Turkey**	IFN-γ, TNF-α, surface molecule expression of CD3+, CD3+ HLA-DR+, CD4+ (T-helper, Th), CD8+ (cytotoxic T lymphocyte), CD19+ (B lymphocyte), CD16+ 56+ (NK cells) and NK cytotoxic activity (NKCC)	43 adolescents with present/lifetime PTSD associated with a history of childhood sexual abuse SA = 33 HC = 10	NA	13–18 years	15.3	Cross-sectional	K-SADS	MDD (K-SADS)	MBI	Flow cytometry	Eosinophil percentage was high (P < 0.05), whereas stimulated intracellular interferon-γ was low (P < 0.05) in adolescents with PTSD-L compared with the control group. In PTSD-P patients exposed to repeated sexual abuse, CD3+ HLA-DR+ T-lymphocyte count was low (P < 0.05) compared with those with one-time sexual abuse.
**(****38****) USA**	CRP, IL-6, TNFR2	702 participants of The Nurses Health Study II SA = 228 HC = 474	90% white race	Three groups:– <11 years - 11–17 years -adulthood	43.9	Cross-sectional	Non-standardized	None	Somoking, physical activity, medication, diet, BMI, alcohol intake	Latex-enhanced immunonephelometric assay ELISA	Levels of CRP and IL-6 were 20%−50% higher in women reporting forced sex than those reporting no sexual abuse.
**(****39****) USA**	Cortisol, MIF	206 high risk youth from low income families with and without prenatal drug exposure moderate/severe SA = 31 HC = 0	12% Caucasian 5% Hispanic 83% African American.	NA	15.3	Cross-sectional	CTQ	None	Prenatal drug exposure	ELISA	Sexual abuse was associated with marginally greater cortisol reactivity, F (5,900) = 2.58, p = .06, when compared to no reported history of sexual abuse.
**(****40****) Germany**	CRP, IL-6, IL-1B, TNF-α, serum oxidative stress markers, PBMC cell composition, mitochondrial flux, mitochondrial density marker	30 health women within 6 days after parturition in the maternity Childhood Abuse = 22 HC = 8	96,8% Caucasian	<18 years	31.6	Cross-sectional	CTQ	HADS	BMI, alcohol use, physical activity, smoking satus	Multiplex (Il1ß, IL-6, TNF),Chemiluminescence (CRP)	Increased levels of TNF, IL-6, IL-1B for individuals with more severe CM experiences (p-values range from 0.07 to 0.08)
**(****41****) Brazil**	Copeptin	136 children selected from a larger research project SA = 7 HC = 71	59,5% Caucasian	6–18 years	9.44	Cross-sectional	Non-standardized	DAWBA	NA	ELISA	Mean differences in copeptin levels between CM+ and CM- groups. Secondary analysis showed that there is no difference regarding specific types of CM regarding copeptin levels (all p >.05).
**(****42****) Germany**	TNF-α, IL-6	214 MMD and 180 HC HC = 180 of which 17 = SA 214 MMD of which 51 = SA	NA	NA	41	Cross-sectional	CTQ	MDD (SCID)	BMI, smoking status, waist rip ratio	ELISA	In the subgroup of traumatized MDD patients, higher severity of childhood sexual abuse was associated with higher levels of both IL-6 and TNF- in a linear fashion.
**(****43****) USA**	CRP, IL-6, IL-1-β, TNF-α	38 healthy subjects CM = 34 SA = NA HC = 4	69,2% Caucasian	NA	35	Cross-sectional	ETI	modified Distress/ Mood Scale	BMI, employment, married, smokers	Multiplex	There was a significant and positive relationship between the number of general traumas and IL-6 levels. There was not a significant direct effect between CSA and inflammatory markers
**(****44****) Brazil**	TNF-α, IFN-γ, IL-2, IL-4, IL-6, IL-10, IL-17A	108 crack cocaine addict woman patients CM = 53 HC = 24	NA	NA	31.3	Longitudinal	CTQ	CSSA BDI ASI-6 SCID	BMI, substance use	Flow cytometry	CM+ showed a large increase in concentration of plasma TNF-α by the third week. The Th1/Th2 ratio on the 18th day of detoxification negatively correlated with the severity of all forms of abuse and neglect and with the severity of withdrawal symptoms by the end of treatment.
**(****45****) China**	120 cytokine expression levels from plasma	64 subjects SA = 3 HC = 22 TDM and CM = 22	Han ethnicity	NA	30	Cross-sectional	CTQ	SDS HAMD	BMI, smoking, medication use	novel protein antibody array methodology	In MDD patients with CM, no significant correlations were found between increased cytokine levels and CTQ total scores or scores of its sub-scales.
**(****46****) USA**	CRP	326 women SA = 45 HC = 204	104 Black 222(68%) White	NA	45.7	Longitudinal	CTQ	MDD (CES-D)	BMI, smoking, medication use	Immunoephelometry	Women who reported exposure to greater emotional abuse, sexual abuse, emotional neglect, and physical neglect as children had elevated CRP levels over the 7 years of follow-up in mid-life
**(****47****) Brazil**	IL-6, TNF-α, IL-10	67 individuals MDD with CM 99 individuals MDD without CM	72% caucasian	NA	25.8	Cross-sectional	CQT	M.I.N.I.	BMI, alcohol abuse, current smoker, psychiatric medication	ELISA	Subjects with childhood trauma showed a significantly higher serum IL-6. Positive correlation between serum IL-6 and physical abuse and emotional abuse. No correlations specifically to CSA.
**(****48****)South Africa**	IL-6, Cortisol	11 children female with suspected sexual abuse SA = 5 Suggestive SA = 6 HC = 0	10 African 1 Indian	10.6	10.6	Cross-sectional	non-standardized (forensic service)	None	NA	Luminex	The residents in the children's home, who had plasma cortisol concentrations below 200 nmol/l, showed increased IL-6 concentrations. In contrast, the relationship between plasma cortisol concentration and IL-6 in the children residing at home showed a suppression of circulating morning IL-6.
**(****49****) Germany**	NF-KB activity, monocyte glucocorticoid sensitivity, and cortisol	36 healthy women Childhood abuse and PTSD = 12 HC = 24	NA	NA	28.75	Cross-sectional	ETI	PSS/DSI	BMI	ELISA	PBMC NF-jB DNA-binding was not associated with ETI total score (rs = 0.23, p = 0.18) or general, physical, or sexual subscale scores
**(****50****)** USA	CRP, IL-6	482 middle-aged male monozygotic and dizygotic twins from the Vietnam Era Twin Registry. SA = 53 HC = 237	NA	<18 years	middle-aged	Cross-sectional	ETI	MDD (SCID)	BMI, blood pressure, smoking, alcohol use, physical activity	Beckman Coulter (CRP), ELISA (IL-6)	Compared to individuals without early trauma, those who were exposed to early trauma were less likely to be married, had higher rates of lifetime history of alcohol abuse or dependence, MDD, and PTSD, as well as higher CRP and IL-6 levels. Levels of inflammation were highest when both twins were exposed to trauma.
**(****51****)USA**	slgA	89 female college students SA = 23 HC = 41	74.2% Caucasian	<14 years	19.24	Cross-sectional	CAS-M	A-SES LEC	NA	ELISA	There was not a significant direct effect of CSA on sIgA. A-SES was significantly higher for the CSA group compared with the non-abused group.
**(****52****)USA**	IL-6, CRP, cortisol	133 pregnant teenagers reported abuse history and depressive symptoms, had two blood draws (second and third trimesters) HC = 0	89.5% Latin	17.8	14–19	Longitudinal	CTQ	MDD (CES-D)	BMI, smokers and history of drug use were excluded	ELISA	Adolescents with more severe abuse and depression had higher IL-6 at the second trimester compared with adolescents with high abuse and low depression
**(****53****)Germany**	IL-6, Fibrinogen, Serum-amyloid A, CRP, adiponectin, TNF-α, resistin, and sE- selectin	25 depressed patients SA = 25 HC = 0	NA	NA	47.8	Cross-sectional(?)	CTQ	HDRS	NA	ELISA	Physical neglect has been significantly associated with increased levels of fibrinogen. There was not a significant direct effect between CSA and inflammatory markers.

*slgA, immunoglobuin A; CRP, C-reactive protein; IL-6, Interleukin-6; IL1-β, interleukin1-β; TNF-α, Tumor necrosis factor-α; IFN-γ, interferon -γ; NF-kB, nuclear factor-kB; MIF, Macrophage Migration Inhibitory Factor; SA, Sexual abuse; HC, Health Controls; CM, Childhood Maltreatment; PTSD, Post Traumatic Stress Disorder; MDD, Major depressive Disorder; M.I.N.I.; Mini International Neuropsychiatric Interview, CAS-M, Child Abuse Survey-Modified; ETI, The early Trauma Inventory; CQT, Childhood Trauma Questionnaire; K-SADS, Schedule for Affective Disorders and Schizophrenia of school age Children; A-SES, Adult Sexual Experience Survey; LEC, Life Events Checklist-Modified; CES-D, Center for Epidemiological Studies Depression Scale; PSS, PTSD Symptom Scale, DSI, Depression Symptom Inventory; HADS, Hospital Anxiety and Depression Scale; HDRS, Hamilton-Depression Rating Scale; CSSA, Cocaine Selective Severity Assessment; BDI, Beck Depression Inventory; ASI-6 Addiction Severity Index; SCID, The Structured Clinical Interview for DSM IV; SDS, Self -rating Depression Scale; HAMD, Hamilton Depression Scale; DAWBA, Development and Well-Being Assessment; PSS, Perceived Stress Scale; CECA, Childhood Experience of Care and Abuse; ELISA, enzyme-linked immunosorbent assay; NA, Not Available*.

### Sexual abuse assessment

The articles presented in this review evaluated sexual abuse as an independent variable alone or a subtype of childhood maltreatment (CM), in this case the authors investigated other types of abuse besides CSA, making correlations between the inflammatory markers and each type of abuse, including CSA. Only two studies evaluated exclusively CSA (37, 48), other two data investigated physical and sexual abuse (38, 51), the remain 13 studies evaluated all subtypes of maltreatment and the correlation of each subtype, and CM global scores, to immune system alterations (39–47, 49, 50, 52, 53).

Nine studies (39, 40, 42, 44–47, 52, 53) used the Childhood Trauma Questionnaire (CTQ), a self-applicable instrument used for assessing physical, emotional, sexual abuses as well as physical and emotional negligence (54). The items are added up so that the five different subtypes of abuse score differently. The clinical cut-off scores are validated and display higher sensitivity and specificity rates (0.85) in comparison to the clinical interview (54, 55). The CTQ psychometric properties present Cronbach's alpha = 0.79–0.94, and reliability coefficients = 0.80–0.83 (56).

Three studies (38, 41, 48) used non-standardized methods to assess CSA. Coelho et al. used face-to-face clinical interviews asking different questions regarding exposure to CM (41). Muller et al. used forensic evaluation to identify whether a child had been abused (48). Another study referred to a violence questionnaire that was mailed to the patients in order to assess physical and sexual abuse in three different stages of life: before 11 years old, between 11 and 17 years old and during adulthood. Although this was not a standardized measurement for CSA, those authors carried out a careful methodological investigation of CSA experiences (38).

Three studies (43, 49, 50) used the Early Trauma Inventory (ETI), another self-applicable instrument containing 56 items to assess childhood maltreatment which investigates 4 different areas: sexual, emotional, physical abuses, and other forms of trauma such as natural disasters, family mental diseases, among others. It also investigates the values, the frequency, the duration and the subjective impacts such as the functional and the emotional ones as well as the impacts on the people's relationships (57). The score obtained could be analyzed separately by attributing points to each type of abuse; or globally by including a total number representing the sum of all the aspects evaluated. The ETI has Cronbach's alpha = 0.75–0.95 (56).

Ayaydin et al. (37) used the PTSD module of the Schedule for Affective Disorders and Schizophrenia for School-Age Children - Present and Lifetime Version (K-SADS-PL) to detect psychological effects of trauma. K-SADS-PL is a semi-structured questionnaire responsible for assessing recent and past diagnosis of children and adolescents following DSM-IV criteria (37).

Waldron et al. (51) used the Child Abuse Survey—Modified (CAS-M) to evaluate CSA. This instrument was adapted from Child Maltreatment Survey and includes 14 items that evaluate physical and sexual abuse by asking the interviewees to rate each item according on a 5-point Likert scale (58).

### Inflammatory markers

Inflammation is an elaborate, finely adjusted process that is controlled at various levels by several distinct systems and processes. Cytokines are essential for the immune system and inflammatory response. Among the cytokines that regulate the inflammatory activity, the stimulating ones are referred to as pro-inflammatory while the inhibiting ones are referred to as anti-inflammatory (59).

The pro-inflammatory cytokines investigated in the included studies were IL-6, IL-1β, TNF- α, soluble fraction of tumor necrosis factor alpha-receptor 2 (TNFR2), and interferon (IFN). Combined, these indicators of immune activity synchronize some cellular functions that promote the inflammation. IL-1, IL-6, e TNF-α stimulates the differentiatiation cytotoxic T cells, which are responsible for the elimination of pathogens harmful for the organism. Inflammatory cytokines also enhance the vascular permeability and engage cell adhesion allowing these defense cells to move from the blood vessels and to migrate to tissues where they neutralize or eliminate pathogens (60).

#### Interleukin-6

Eleven studies evaluated the correlation between IL-6 and CSA with heterogeneous results (38–41, 43–45, 50, 51, 53, 55). Walsh et al. evaluated 133 pregnant teenagers with sexual abuse history on their second and third trimesters. Adolescents with history of more severe abuse and depression had higher IL-6 levels in the second trimester compared to those with less severe abuse and milder depression (52). Bertone-Johnson et al. investigated IL-6 levels in 702 participants of the Nurses' Health Study II, of whom 228 had a child sexual abuse history. The IL-6 values obtained were 20–50% higher among women who reported CSA than among the ones who haven't suffered any kind of abuse (38). In addition to that, Boeck et al. investigated the association between oxidative stress and alterations on the mitochondria functions considering the inflammation ascertained in individuals with history of childhood maltreatment. Thirty women presenting different levels of abuse and negligence severity were evaluated. There was a significant marginal association of CTQ classification with the spontaneous release of IL-6 by peripheral blood mononuclear cells (PBMCs), with increased IL-6 levels in individuals with more severe CM experiences (40). Furthermore, Grosse et al. compared 214 patients with major depressive disorder (MDD) and 180 controls; of these, 51 and 17 participants, respectively, reported sexual abuse. The authors also indicate the severity of the mistreatment they were victims of by dividing them into 4 different stages ranging from “none or minimum” to “severe or extreme.” Results showed a strong association between the severity reported and the increase on the IL-6 levels on MDD patients victims of CSA (42). In addition, Levandowiski et al. assessed immunoendocrine parameters of 108 female crack cocaine users in three different periods of a detox treatment. They showed higher IL-6 levels in the childhood maltreated group when compared to the controls and to the patients with no history of maltreatment in all assessments (44). Finally, Muller et al. assessed 11 girls, of whom 5 had been sexually abused and 6 had experienced suggestive sexual assault; 5 of the girls lived at home and 6 had been placed in a children's shelter. The authors found an inverse correlation between cortisol and IL-6: the residents of the children's shelter displayed plasma cortisol concentrations inferior to 200 nmlo/L in spite of the enhance of the IL-6 levels. Inversely, no correlation was observed among the girls residing at their own home (48).

The others five studies did not find association between CSA and IL-6 levels. Hartwell et al. demonstrated an association between the number of traumatic events suffered and the IL-6 levels in a healthy population; however, there was no significant direct effect on CSA and inflammatory markers (43). Rooks et al. Also measured IL-6 to determine inflammation among 482 middle-aged male twins (241 pairs) from the Vietnam Era Twin Registry, 53 of whom reported CSA. The authors concluded that the twins exposed to early trauma presented higher levels of IL-6 compared to those who did not experience any early trauma; however, the results for correlation between CSA and IL-6 levels were unclear (50). Lu et al. recruited 22 depressive patients exposed to childhood trauma, 22 depressive without any trauma history and 22 controls, and found no correlation of IL-6 levels and depression or early trauma. They hypothesized that the current antidepressive treatment had been able to normalize the cytokines levels (45). Zeugmann et al. retrospectively evaluated 25 depressed patients with childhood maltreatment history and found no correlation between CSA and IL-6 levels (53). Pedrotti Moreira et al. found no correlations between CSA and IL-6, although they found an association between physical and emotional abuse and higher IL-6 levels (47).

#### Tumor necrosis factor- α

Seven studies analyzed the levels of TNF-α, a cytokine produced by macrophages and natural killer cell, which presented rather conflicting results (37, 40, 42–44, 47, 53). Grosse et al. demonstrated increased TNF-α levels in patients with MDD who reported CSA, as compared to controls (β = 0.570, *p* = 0.001) (51). Boeck et al. analyzed pro-inflammatory markers and serum oxidative stress levels in blood samples collected from 30 women who reported childhood abuse history; they found an association between CM and the increase on the levels of pro-inflammatory cytokines, indicating that the PBMC culture reveals a higher propensity to the spontaneous release of higher levels of TNF-α (40). Hartwell et al. referred to ETI to investigate correlations between CM and inflammatory markers in 38 participants, which demonstrated that the number of traumatic events throughout life was closely related with an increase on the TNF-α levels; however, when considering trauma subscales, there was no association between CSA and TNF-α levels (43). Similarly, Ayaydin et al. evaluated 43 adolescents, 33 of whom reported having history of CSA, and have not demonstrated any significant differences on the TNF-α levels when comparing participants with CSA to healthy controls (37). Levandowski et al. showed an increase on the TNF-α levels in childhood maltreated group after 11 days of detoxification treatment, compared to the controls and to the non-maltreated group (44). Zeugman et al. and Pedrotti Moreira et al. found no correlations between CSA and TNF-α levels (47, 53).

#### Interleukin−1β

Two studies investigated the association between CSA and IL-1β (40, 43). Hartwell et al. indicated that the number of traumatic events was positively associated with the levels of IL1- β. However, such associations did not remain in regression analyses and there was no positive association between CSA and IL-1β levels (43). Boeck et al. also concluded that a higher level of IL-1β was associated with CTQ classification, with increased IL-1β levels among individuals with more severe cases of CM (40).

#### C-reactive protein

Seven studies investigated CRP, which is an acute phase protein, with three indicating increase in CRP levels associated with CSA (38, 40, 43, 46, 50, 52, 53). Walsh et al. showed a significant association between levels of CRP and higher pre-pregnancy body-mass-index (BMI) in pregnant adolescents, in the second and third trimesters, which had experienced child sexual abuse. However, abuse and depression did not interact to predict CRP at two time points (52). Matthews et al. used the CTQ to retrospectively assess abuse and neglect in childhood and adolescence among 326 women who underwent CRP measurement over seven annual visits. Women victims of sexual or emotional abuses or victims of physical or emotional negligence throughout childhood showed increase on the CRP levels over the 7 year follow-up. Significant indirect effects on the CRP levels were observed when considering the BMI values and 111.3% of the effect of sexual abuse was mediated by BMI (46). Bertone-Johnson et al. analyzed CRP in 702 women, of which 32% reported CSA. The authors found elevated levels of CRP in those reporting history of unwanted touching (0.92 mg/L) and forced sex (1.02 mg/L) in adolescence, compared to 0.68 mg/L in women without any history of abuse as children (*p* = 0.04) (38). In addition, Rooks et al. found increased CRP levels correlated to the total trauma score, and twins with early trauma had 22% higher CRP levels (50). On the other hand, Boeck et al. demonstrated generally low levels of CRP, mainly in participants with severe CM experiences (38). Additionally, Zeugmann et al. and Hartwell et al. was unable to establish any associations between the CSA and CRP levels (43, 53).

#### Interferon- γ

Two studies analyzed the correlation between interferon γ (IFN-γ) levels and CSA (37, 44). Ayaydin et al. demonstrated that stimulated IFN-γ was significantly lower in patients with present and lifetime PTSD who were exposed to repeated sexual abuse, compared to controls (37); in turn, Levandowski et al. found lower levels of IFN- γ compared to controls, but those levels showed an increase curve during the detoxification days (44).

#### Nuclear factor -kB

Only one study (Pace et al.) assessed NF-kB in PBMCs obtained from 12 different women who suffer from PTSD deriving from the childhood abuse and 24 different controls; no association was found between PBMC NF-kB DNA binding and sexual abuse subscale scores, but PBMC obtained from those women who reported having a history of abuse showed increased Nf-kB DNA-binding values compared to controls ones (49).

#### Macrophage migration inhibitory factor

Solely one research studied MIF. Bick et al. examined the HPA axis and the signs of inflammation in 206 teenagers who reported having prenatal exposure to psychoactive substances and childhood maltreatment. The authors evaluated levels of cortisol and macrophage migration inhibitory factor (MIF). Results have indicated that reports of physical neglect, but no other subtypes of CM, are significantly associated with cortisol and MIF values (39).

#### Copeptin

Merely one study investigated Copeptin. Coelho et al. evaluated 136 children; 56 experienced CM and 7 reported sexual abuse. The authors found that serum levels of copeptin were significantly higher in children exposed to CM, but secondary analyses indicated no differences for specific types of CM regarding copeptin levels (41).

#### Immunoglobulin A

Only one study evaluated slgA. Waldron et al. examined the impact of CSA on salivary slgA levels. They evaluated 89 female college students, 23 of whom reporting CSA. The results indicated no significant direct effect of CSA on slgA, but the relationship between CSA and slgA was mediated by scores on the Adult Sexual Experiences Survey, indicating that the sexual traumas these individuals were subjected to during adulthood were predictive of the slgA levels (51).

#### Receptor 2 TNF-α

Solely the Bertone-Johnson et al. study, compared levels of immune activity in women who reported CSA and/or physical abuse to the levels observed in healthy controls. The authors found higher levels of receptor 2 TNF-α among women reporting sexual and physical abuses during adolescence. However, these results were not significant enough and the relevance of such comparisons was limited (38).

## Discussion

Despite the novelty of studies on the correlation of inflammation with CSA, our review found 17 studies, involving 2,723 participants, which specifically evaluated the relationship of CSA and inflammation. Five studies evaluated children victims of recent abuse (37, 39, 41, 48, 52), one evaluated biological markers in young adults with mean age of 19 (51), and the remaining eleven studies evaluated chronic consequences of sexual abuse in adults (38, 40, 42–47, 49, 50). All five studies that focused on recent abuse found positive correlations between biomarkers and CM, regardless the occurrence of sexual abuse.

Most studies analyzed the long-term effects of CSA on pro-inflammatory cytokines IL-6, IL-1β, TNF-α, and CRP levels, showing an increase on inflammatory activity during adult life associated with the presence of CM. In general, there was a positive correlation between CM and pro-inflammatory cytokine concentrations. Increased numbers of maltreatment events was associated with higher concentrations of proinflammatory cytokines; however, when the effect of sexual abuse during childhood was specifically analyzed, this correlation was not observed. We speculate that some confounders, such as the presence of psychiatric disorder comorbidity (e.g., MDD and PTSD) and the length of time of abuse or number of traumatic events, could be related to glucocorticoid resistance and, consequently, interfered with the production of cytokines. Therefore, studies that assessed chronic PTSD patients would be likely to display different results when compared to studies that assessed patients who have just experienced trauma.

Consistent researches indicate that immune system alterations are present in several mental disorders such as mood disorders, bipolar disorder, schizophrenia, and even autism spectrum, showing that increased inflammatory markers are clearly related to mental disorders (61). One of the most important hypothesis concerns the microglial activation, which are brain cells responsible for neuroiflammation in response to ambient changes and brain damage (62, 63). Furthermore, peripheral inflammatory markers are related to the induction of psychiatric symptoms (64). It is important to notice that the increase on inflammatory markers are unspecific for mental disorders and are present in several psychiatric disorders.

The association of CSA with psychiatry disorders in adult life is well established in literature and some disorders are associated with increased inflammatory marker levels regardless any CM history, particularly mood disorders and PTSD (27, 65). Therefore, we raised the question about which factor; either CSA or psychiatric disorder has greater influence on the immune system. In this review seven studies analyzed a population without any previous psychiatric diagnosis in axis I (38, 40, 41, 43, 46, 48, 51). Of the aforementioned studies, five studies evaluated inflammatory alterations in adults (38, 40, 43, 46, 51), and two investigated inflammatory alterations at childhood (41, 48). Although the sample didn't count on axis I diagnosis, most studies found a positive association with psychiatric symptoms in scales results, as expected. Three studies (38, 40, 46) showed a significant increase in CRP, IL-6, and TNF-α levels in healthy adults with CSA history. These results are limited to a few studies on CSA until now. A recent study conducted by Do Prado et al. ascertains that the effects of CM on the immune and endocrine pathways of teenagers with no history of psychiatric symptoms and indicated the presence of pro-inflammatory activity in those healthy ones who were exposed to childhood abuse (66). Nevertheless, it could be a way to better understand if CSA only predicts adult indicators of immune function when an established psychiatric disorder occurs; or whether CSA increases inflammatory levels regardless the outset of the illness thus contributing for the increase on the susceptibility to mental disorders throughout life.

There are evident gender variations in biological systems related to stress response (67). Previous studies assessing specific effects of the early life trauma related to gender on the stress system showed alterations in HPA axis with increased corticotrophin and cortisol response in women (68, 69). These variables could influence the levels of inflammatory markers in the organism. Of the studies compiled in this review, only one examined a male sample composed by twins, demonstrating a possible familial factor associated with adulthood inflammation (50).

Of the other 16 studies that included female in the sample, only five covered the information about what menstrual cycle phase the women were in (38, 40, 46, 49, 52). Despite the fact that this information is relevant for the results, each study measured the inflammatory level in a different phase: Bertone-Johnson et al. (38) collected blood during the menstrual cycle; Boeck et al. (40) examined women in a follow up 3 months postpartum; Walsh et al. (52) studied pregnant teenagers; Matthews et al. (42) studied women on menopause and Pace et al. (49) evaluated women in luteal phase. Nevertheless, it is important to emphasize the confounding factor represented by these measurement in different phases, or even the studies that did not evaluate which menstrual cycle phase these women were in, which could influence the results since the biological influence of gender and sexual hormones in HPA axis is well known.

CSA has a high prevalence of co-occurrence and correlation with multiple types of child maltreatment, therefore considering sexual abuse as an isolate variable may be a confounding factor in this review. A recent study examining the correlations among types of maltreatment shows that some classifications underestimate the co-occurrence indexes among the several types of maltreatment. This indicates it is common and that the physical and emotional abuse were the subtypes with higher rate of concomitance (70). In this sense, the official classification may neither represent the experience as accurately as expected in the case of children victims of abuse nor take the several types of maltreatment into account. Nevertheless, most of the studies compiled in this review investigated more than one subtype of maltreatment and they did not examine the concomitant occurrence of its various subtypes. There are few studies focusing only in CSA, it is more common evaluate the maltreatment by instruments and analyse the scales to identify the subtypes. Although there are different ways to evaluate CM and its subtypes, there is evidence that when maltreatment subtypes are studied separately, it impacts differently on each inflammatory marker (33). Evaluating separately each type of maltreatment and then their co-occurrence, taking into consideration the severity and age of maltreatment as well as patterns of recurrence, may be more accurate to understand the biological consequences of abuse instead of considering maltreatment as one isolated phenomenon since its subtypes co-exist. In this review, Boeck et al. conducted a classification of maltreatment corresponding to severity, showing an augmented oxidative stress level with higher maltreatment load (40). Furthermore, Pechtel et al. compared amygdala volume in adults with CM to the control group showing that the right amygdala's volume varied 27% according to the severity of the trauma the individual was exposed to also suggesting that the subtype and severity of maltreatment have specific neurobiological consequences (71). Some reviews demonstrated that CSA could be considered as one of the most severe forms of trauma (72). In this sense, the biological implications of this trauma subtype may be different than others not as grievous.

Several variables could also be associated with CSA and with alterations on immune activity. The increase in body mass index and unhealthy behaviors that elevate the risk of infection such as drug use, risky sex or low-grade inflammation such as smoking; are some examples. Most studies covered in this review evaluated some of these factors, however, the most frequent one was body mass index, followed by smoking, physical activity, drug use, and others less common, such as blood pressure status and waist hip ratio. All these factors are associated to inflammatory alterations, but most studies conducted did not correlate them directly to immune alterations or even CSA. The association between lifestyle factors, CM and inflammatory markers was previously stated (73) and CSA is a widely-known risk factor for obesity (74). The changes in HPA axis mediating the alteration process in immune system could explain this strong correlation. More studies investigating the epigenetic factors involved in these correlations would help to clarify the biological mechanism that connects child sexual abuse to obesity, inflammation and consequently to physical disease.

Nine of the 17 studies did not mention the victim's age when abused (39, 42–47, 49, 53), which is also a failure since there is a neuroplasticity in childhood, the period in which the brain structures are in maturity and there is evidence that earlier exposure to adversity may have serious health consequences compared to later experiences (75). Blanco et al. evaluated neurological changes in brain structure of individuals with CSA showing irregularities in cortical and subcortical regions in abused victims. In addition, the age of the occurrence of CSA seemed to be associated with different neurological brain structures, particularly with hippocampal volume reduced among CSA ages 3–5 and 11–13 (76). Most studies presented in this review evaluated CSA in adolescents and the minimum age was six, therefore, the studies about CSA may be missing an important period of cerebral development and the consequences of early abuse have not been reported properly.

Another aspect to be noticed is the choice of instruments for assessment of trauma used in the studies, although there seems to be a preference for using the CTQ, used by eight studies, accounting for half of the studies. Previous studies related to CTQ displayed its validity and reliability in the case of both healthy controls and patients (77). However, other instruments were applied, some of which included other early life stress subtypes. Despite the lack of consensus on the gold standard instrument to collect data about childhood abuse, CTQ and ETI appear to be more accurate than others not validated.

Memory recall for collecting retrospective data of abuse has a risk of bias in all studies that collect childhood experiences in adults; it is inaccurate, can change over subsequent assessments and may not correlate with more objective measurements. Furthermore, the traumatic experiences on their own can modify the information contained on the individuals' memories as well as their meanings. The instruments used for the regression analysis depend on the memory people have of the traumas they were exposed to, so it can be considered an important limitation of the studies that evaluated childhood trauma in adults.

The design of the studies is another matter to be analyzed. Most studies had a cross-sectional design and because of that, it was not possible to assess the influence exercised by time on the inflammatory markers after the CSA. More studies with longitudinal design may elucidate several questions about correlation of CSA and immune changes, what time it occurs and its relationship with other pathologies.

The presence of conflicting results, usually present in studies of biomarkers in trauma and diagnosed PTSD is a consequence of the complexity of this subject. CM in the form of neglect and abuse has different impacts on biological activity that are dependent on chronicity, repeated traumas, and the age of occurrence. The immune and the stress response systems, including the HPA axis and sympathetic nervous system, are integrated and reactive to the environment. In this way, upon selecting the sample population it is fundamental to distinguish differences by gender, chronicity, and stage of development. The same rationale exists for the type of abuse. Specifically, sexual abuse has different epidemiology insomuch that it has long term consequences, occurs more frequently to girls, and is usually perpetrated by a person or relative close to the victim. According to our results, most studies have found correlations between CM and inflammatory markers, but the correlation is not maintained when specifically considering CSA. A low number of trauma subtypes in the sample and the confounder factors aforementioned could explain these discrepant results.

We acknowledge some limitations of the present study. Firstly, there was considerable heterogeneity in the methods used to evaluate CSA, the majority of the studies found in the databases investigated maltreatment globally and did not analyse the effect of the subtypes of CM experiences on inflammatory markers, and only a few authors analyse CM subscales. All of this is a limiting factor since we could not include more studies in this review, because the presence of CSA was not clear in others studies. Moreover, some methods are not standardized and memory recall for collecting retrospective data of abuse poses as a risk of bias in all studies that use instruments to collect childhood experiences in adults. Secondly, most studies have small sample and effect sizes. Further on, as we followed PRISMA guidelines, we did not use a quality assessment tool to evaluate each study included in the review; in addition to that, research in the area of validation and quality of studies have demonstrated heterogeneous results in the systematic reviews area (78). Due to the limitations of the studies identified above it was not possible to perform a meta-analysis, therefore our analysis was only qualitative rather than quantitative.

Prevention programs of CSA with information and educational committee with a particular focus on vulnerable populations, could have a noteworthy positive result since CSA is an important risk factor for the development of many psychiatric and other medical diseases. Further on, the CSA influences on the inflammatory system are also mediated by these pathological conditions. Studies evaluating the correlation of CSA with inflammatory markers could play a key role on the prevention of mental disorders or even physical ones. Further studies are needed to understand the neurobiological impact of CSA, including the changes in HPA axis activity, immune system, and epigenetic marks. Prospective, long-term, and global studies are necessary for a more comprehensive understanding of the psychological and biological consequences of this prevalent and devastating event.

## Author contributions

AD and CM contributed to perform the search, data collection, screening and write the article. JN helped writing and reviewing the manuscript. MM and MJ participated in the study design and contributed reviewing the manuscript. AM contributed writing and reviewing the manuscript. All authors have read and approved this final version.

### Conflict of interest statement

MM is supported by Scholarship CNPq, funded by FAPESP (grant 2014/12559-5). MJ is supported by Scholarship CNPq, funded by FAPESP (grant 2014/12559-5) and is a Newton International Fellow of the Academy of Medical Sciences and the Royal Society, UK, funded by the Biomedical Research Centre (BRC), a partnership of South London and Maudsley NHS Foundation Trust and the Institute of Psychiatry, Psychology and Neuroscience (IoPPN) at King's College London. The remaining authors declare that the research was conducted in the absence of any commercial or financial relationships that could be construed as a potential conflict of interest.

## References

[1] Martins CMS Tofoli SMC Baes CW Juruena M Analysis of the occurrence of early life stress in adult psychiatric patients: a systematic review. Psychol Neurosci. (2011) 4:219–27. 10.3922/j.psns.2011.2.007

[2] Danese A Pariante CM Caspi A Taylor A Poulton R. Childhood maltreatment predicts adult inflammation in a life-course study. Proc Natl Acad Sci USA. (2007) 104:1319–24. 10.1073/pnas.0610362104 17229839 PMC1783123

[3] Edwards VJ Holden GW Felitti VJ Anda RF. Relationship between multiple forms of childhood maltreatment and adult mental health in community respondents: results from the adverse childhood experiences study. Am J Psychiatry (2003) 160:1453–60. 10.1176/appi.ajp.160.8.1453 12900308

[4] Mello AF Mello MF Carpenter LL Price LH. Update on stress and depression: the role of the hypothalamic-pituitary-adrenal (HPA) axis. Rev Bras Psiquiatr. (2003) 25:231–8. 15328550 10.1590/s1516-44462003000400010 PMC4479183

[5] Mello MF Faria AA Mello AF Carpenter LL Tyrka AR Price LH. Childhood maltreatment and adult psychopathology: pathways to hypothalamic-pituitary-adrenal axis dysfunction. Rev Bras Psiquiatr. (2009) 31(Suppl. 2):S41–8. 10.1590/S1516-44462009000600002 19967199 PMC4476494

[6] Rich-Edwards JW Spiegelman D Lividoti Hibert EN Jun HJ Todd TJ Kawachi I. Abuse in childhood and adolescence as a predictor of type 2 diabetes in adult women. Am J Prev Med. (2010) 39:529–36. 10.1016/j.amepre.2010.09.007 21084073 PMC3003936

[7] Wells D Guidelines for Medico-Legal Care for Victims of Sexual Violence. World Health Organization (2003).

[8] Ezzati M Lopez AD Rodgers A Murray CJL Comparative quantification of health risks: global and regional burden of diseases attributable to selected major risks. Comp Quantif Heal Risks Glob Reg Burd Dis Attrib to Sel Major Risk Factors (2004) 1:i–xxiv. 10.1016/j.amepre.2004.07.014

[9] Stoltenborgh M van Ijzendoorn MH Euser EM Bakermans-Kranenburg MJ. A global perspective on child sexual abuse: meta-analysis of prevalence around the world. Child Maltreat. (2011) 16:79–101. 10.1177/1077559511403920 21511741

[10] Gill JM Saligan L Woods S Page G. PTSD is associated with an excess of inflammatory immune activities. Perspect Psychiatr Care (2009) 45:262–77. 10.1111/j.1744-6163.2009.00229.x 19780999

[11] Juruena MF. Early-life stress and HPA axis trigger recurrent adulthood depression. Epilepsy Behav. (2014) 38:148–59. 10.1016/j.yebeh.2013.10.020 24269030

[12] De Kloet ER Rots NY Cools AR. Brain-corticosteroid hormone dialogue: slow and persistent. Cell Mol Neurobiol. (1996) 16:345–56. 10.1007/BF02088100 8818401 PMC11563134

[13] Sapolsky R Rivier C Yamamoto G Plotsky P Vale W. Interleukin-1 stimulates the secretion of hypothalamic corticotropin-releasing factor. Science (1987) 238:522–4. 10.1126/science.2821621 2821621

[14] Irwin MR Cole SW. Reciprocal regulation of the neural and innate immune systems. Nat Rev Immunol. (2011) 11:625–32. 10.1038/nri3042 21818124 PMC3597082

[15] Schleimer RP. An overview of glucocorticoid anti-inflammatory actions. Eur J Clin Pharmacol. (1993) 45(Suppl 1):S3–7. 10.1007/BF01844196 8313932

[16] Marques AH Silverman MN Sternberg EM. Glucocorticoid dysregulations and their clinical correlates: from receptors to therapeutics. Ann NY Acad Sci.(2009) 1179:1–18. 10.1111/j.1749-6632.2009.04987.x 19906229 PMC2933142

[17] Slavich GM Irwin MR. From stress to inflammation and major depressive disorder: a social signal transduction theory of depression. Psychol Bull. (2014) 140:774–815. 10.1037/a0035302 24417575 PMC4006295

[18] Camacho-Arroyo I López-Griego L Morales-Montor J. The role of cytokines in the regulation of neurotransmission. Neuroimmunomodulation (2009) 16:1–12. 10.1159/000179661 19077440

[19] Anisman H Merali Z, Cytokines stress and depressive illness. Brain Behav Immun. 16:513–24. 10.1016/S0889-1591(02)00009-0 12401465

[20] Hart B Biological basis of hte behavior of sick animals. Neurosci Biobehav Rev. (1988) 12:123–37. 10.1016/S0149-7634(88)80004-6 3050629

[21] Goodwin RD Stein MB. Association between childhood trauma and physical disorders among adults in the United States. Psychol Med. (2004) 34:509–20. 10.1017/S003329170300134X 15259836

[22] Lopes RP Grassi-Oliveira R de Almeida LR Stein LM Luz C Teixeira AL. Neuroimmunoendocrine interactions in patients with recurrent major depression, increased early life stress and long-standing posttraumatic stress disorder symptoms. Neuroimmunomodulation (2012) 19:33–42. 10.1159/000327352 22067620

[23] Carpenter LL Gawuga CE Tyrka AR Lee JK Anderson GM Price LH. Association between plasma IL-6 response to acute stress and early-life adversity in healthy adults. Neuropsychopharmacology (2010) 35:2617–23. 10.1038/npp.2010.159 20881945 PMC2978751

[24] Danese A Moffitt TE Pariante CM Ambler A Poulton R Caspi A. Elevated inflammation levels in depressed adults with a history of childhood maltreatment. Arch Gen Psychiatry (2008) 65:409–16. 10.1001/archpsyc.65.4.409 18391129 PMC2923056

[25] Cougle JR Timpano KR Sachs-Ericsson N Keough ME Riccardi CJ. Examining the unique relationships between anxiety disorders and childhood physical and sexual abuse in the National Comorbidity Survey-Replication. Psychiatry Res. (2010) 177:150–5. 10.1016/j.psychres.2009.03.008 20381878

[26] Varese F Drukker M Lieverse R Lataster T Viechtbauer W Read J Van Os J. Childhood adversities increase the risk of psychosis: a meta-analysis of patient-control, prospective- and cross-sectional cohort studies. Schizophr Bull. (2012) 38:661–71. 10.1093/schbul/sbs050 22461484 PMC3406538

[27] Carr CP Martins CMS Stingel AM Lemgruber VB Juruena MF. The role of early life stress in adult psychiatric disorders: a systematic review according to childhood trauma subtypes. J Nerv Ment Dis. (2013) 201:1007–20. 10.1097/NMD.0000000000000049 24284634

[28] Kimerling R Alvarez J Pavao J Kaminski A Baumrind N. Epidemiology and consequences of women's revictimization. Women Heal Issues (2007) 17:101–6. 10.1016/j.whi.2006.12.002 17403467

[29] Luz MP Coutinho ESF Berger W Mendlowicz MV Vilete LMP Mello MF. Conditional risk for posttraumatic stress disorder in an epidemiological study of a Brazilian urban population. J Psychiatr Res. (2016) 72:51–7. 10.1016/j.jpsychires.2015.10.011 26540404

[30] Chen LP Murad MH Paras ML Colbenson KM Sattler AL Goranson EN. Sexual abuse and lifetime diagnosis of psychiatric disorders: systematic review and meta-analysis. Mayo Clin Proc. (2010) 85:618–29. 10.4065/mcp.2009.0583 20458101 PMC2894717

[31] Bebbington PE Cooper C Minot S Brugha TS Jenkins R Meltzer H. Suicide attempts, gender, and sexual abuse: data from the 2000 British psychiatric morbidity survey. Am J Psychiatry (2009) 166:1135–40. 10.1176/appi.ajp.2009.09030310 19723788

[32] Maniglio R. Child sexual abuse in the etiology of depression: a systematic review of reviews. Depress Anxiety (2010) 27:631–42. 10.1002/da.20687 20336807

[33] Baumeister D Akhtar R Ciufolini S Pariante CM Mondelli V. Childhood trauma and adulthood inflammation. a meta-analysis Peripher C-reactive protein, interleukin-6 tumour necrosis factor-α. Mol Psychiatry (2015) 21:642–9. 10.1038/mp.2015.67 26033244 PMC4564950

[34] Nemeroff CB. Paradise lost: the neurobiological and clinical consequences of child abuse and neglect. Neuron (2016) 89:892–909. 10.1016/j.neuron.2016.01.019 26938439

[35] Heim CM Mayberg HS Mletzko T Nemeroff CB Pruessner JC. Decreased cortical representation of genital somatosensory field after childhood sexual abuse. Am J Psychiatry (2013) 170:616–23. 10.1176/appi.ajp.2013.12070950 23732967

[36] Moher D Liberati A Tetzlaff J Altman DG. Preferred reporting items for systematic reviews and meta-analyses: the PRISMA statement. Int J Surg. (2010) 8:336–41. 10.1016/j.ijsu.2010.02.007 20171303

[37] Ayaydin H Abali O Akdeniz NO Kok BE Gunes A Yildirim A. Immune system changes after sexual abuse in adolescents. Pediatr Int. (2016) 58:105–12. 10.1111/ped.12767 26224367

[38] Bertone-Johnson ER Whitcomb BW Missmer SA Karlson EW Rich-Edwards JW. Inflammation and early-life abuse in women. Am J Prev Med. (2012) 43:611–20. 10.1016/j.amepre.2012.08.014 23159256 PMC3504353

[39] Bick J Nguyen V Leng L Piecychna M Crowley MJ Bucala R. Preliminary associations between childhood neglect, MIF, and cortisol: potential pathways to long-term disease risk. Dev Psychobiol. (2015) 57:131–9. 10.1002/dev.21265 25380347 PMC4337818

[40] Boeck C Koenig AM Schury K Geiger ML Karabatsiakis A Wilker S. Inflammation in adult women with a history of child maltreatment: the involvement of mitochondrial alterations and oxidative stress. Mitochondrion (2016) 30:197–207. 10.1016/j.mito.2016.08.006 27530300

[41] Coelho R Levandowski ML Mansur RB da Cunha GR Asevedo E Zugman A. Serum copeptin in children exposed to maltreatment. Psychiatry Clin Neurosci. (2016) 70:434–41. 10.1111/pcn.12412 27278269

[42] Grosse L Ambrée O Jörgens S Jawahar MC Singhal G Stacey D Cytokine levels in major depression are related to childhood trauma but not to recent stressors. Psychoneuroendocrinology (2016) 73:24–31. 10.1016/j.psyneuen.2016.07.205 27448525

[43] Hartwell KJ Moran-Santa Maria MM Twal WO Shaftman S DeSantis SM McRae-Clark AL. Association of elevated cytokines with childhood adversity in a sample of healthy adults. J Psychiatr Res. (2013) 47:604–10. 10.1016/j.jpsychires.2013.01.008 23415658 PMC3594625

[44] Levandowski ML Viola TW Prado CH Wieck A Bauer ME Brietzke E. Distinct behavioral and immunoendocrine parameters during crack cocaine abstinence in women reporting childhood abuse and neglect. Drug Alcohol Depend. (2016) 167:140–8. 10.1016/j.drugalcdep.2016.08.010 27530287

[45] Lu S Peng H Wang L Vasish S Zhang Y Gao W. Elevated specific peripheral cytokines found in major depressive disorder patients with childhood trauma exposure: a cytokine antibody array analysis. Compr Psychiatry (2013) 54:953–61. 10.1016/j.comppsych.2013.03.026 23639406

[46] Matthews KA Chang YF Thurston RC Bromberger JT. Child abuse is related to inflammation in mid-life women: role of obesity. Brain Behav Immun. (2014) 36:29–34. 10.1016/j.bbi.2013.09.013 24076375 PMC3947183

[47] Pedrotti Moreira F Wiener CD Jansen K Portela LV Lara DR Souza LD de M. Childhood trauma and increased peripheral cytokines in young adults with major depressive: population-based study. J Neuroimmunol. (2018) 319:112–6. 10.1016/j.jneuroim.2018.02.018 29519722

[48] Muller D Errington S Szabo C Pitts N Jacklin L Cortisol and IL-6 Responses to stress in female children presenting at a sexual abuse clinic. J Child Adolesc Trauma (2014) 7:185–91. 10.1007/s40653-014-0019-7

[49] Pace TW Wingenfeld K Schmidt I Meinlschmidt G Hellhammer DH Heim CM. Increased peripheral NF-kappaB pathway activity in women with childhood abuse-related posttraumatic stress disorder. Brain BehavImmun. (2012) 26:13–7. 10.1016/j.bbi.2011.07.232 21801830

[50] Rooks C Veledar E Goldberg J Bremner JD Vaccarino V. Early trauma and inflammation: role of familial factors in a study of twins. Psychosom Med. (2012) 74:146–52. 10.1097/PSY.0b013e318240a7d8 22286843 PMC3307790

[51] Waldron JC Scarpa A Kim-Spoon J Coe CL. Adult sexual experiences as a mediator between child abuse and current secretory immunoglobulin A levels. J Interpers Violence (2016) 31:942–60. 10.1177/0886260514556763 25395225

[52] Walsh K Basu A Werner E Lee S Feng T Osborne LM. Associations among child abuse, depression, and interleukin-6 in pregnant adolescents: paradoxical findings. Psychosom Med. (2016) 78:920–30. 10.1097/PSY.0000000000000344 27187846 PMC5067964

[53] Zeugmann S Buehrsch N Bajbouj M Heuser I Anghelescu I Quante A. Childhood maltreatment and adult proinflammatory status in patients with major depression. Psychiatr Danub. (2013) 25:227–35. 24048389

[54] Bernstein DP Stein JA Newcomb MD Walker E Pogge D Ahluvalia T. Development and validation of a brief screening version of the Childhood Trauma Questionnaire. Child Abus Negl. (2003) 27:169–90. 10.1016/S0145-2134(02)00541-0 12615092

[55] Walker EA Gelfand A Katon WJ Koss MP Von Korff M Bernstein D. Adult health status of women with histories of childhood abuse and neglect. Am J Med. (1999) 107:332–9. 10.1016/S0002-9343(99)00235-1 10527034

[56] Roy CA Perry JC. Instruments for the assessment of childhood trauma in adults. J Nerv Ment Dis. (2004) 192:343–51. 10.1097/01.nmd.0000126701.23121.fa 15126888

[57] Bremner JD Vermetten E Mazure CM. Development and preliminary psychometric properties of an instrument for the measurement of childhood trauma: the Early Trauma Inventory. Depress Anxiety (2000) 12:1–12. 10.1002/1520-6394(2000)12:1<1::AID-DA1>3.0.CO;2-W 10999240

[58] Yang B Clum GA. Life Stress, social support, and problem-solving skills predictive of depressive symptoms, hopelessness, and suicide ideation in an asian student population: a test of a model. Suicide Life-Threatening Behav. (1994) 24:127–39. 10.1111/j.1943-278X.1994.tb00797.x 8053007

[59] Curfs JHAJ Meis JFGM Hoogkamp-Korstanje JAA. A primer on cytokines: sources, receptors, effects, and inducers. Clin Microbiol Rev. (1997) 10:742–80. 9336671 10.1128/cmr.10.4.742 PMC172943

[60] Dhabhar FS Malarkey WB Neri E McEwen BS. Stress-induced redistribution of immune cells-from barracks to boulevards to battlefields: a tale of three hormones - curt richter award winner. Psychoneuroendocrinology (2012) 37:1345–68. 10.1016/j.psyneuen.2012.05.008 22727761 PMC3412918

[61] Réus GZ Fries GR Stertz L Badawy M Passos IC Barichello T. The role of inflammation and microglial activation in the pathophysiology of psychiatric disorders. Neuroscience (2015) 300:141–54. 10.1016/j.neuroscience.2015.05.018 25981208

[62] Nimmerjahn A Kirchhoff F Helmchen F Neuroscience: resting microglial cells are highly dynamic surveillants of brain parenchyma *in vivo*. Science (2005) 308:1314–8. 10.1126/science.1110647 15831717

[63] Stertz L Magalhães PVS Kapczinski F. Is bipolar disorder an inflammatory condition? the relevance of microglial activation. Curr Opin Psychiatry (2013) 26:19–26. 10.1097/YCO.0b013e32835aa4b4 23196997

[64] Dantzer R O'Connor JC Freund GG Johnson RW Kelley KW. From inflammation to sickness and depression: when the immune system subjugates the brain. Nat Rev Neurosci. (2008) 9:46–56. 10.1038/nrn2297 18073775 PMC2919277

[65] Dowlati Y Herrmann N Swardfager W Liu H Sham L Reim EK. A Meta-analysis of cytokines in major depression. Biol Psychiatry (2010) 67:446–57. 10.1016/j.biopsych.2009.09.033 20015486

[66] Do Prado CH Grassi-Oliveira R Daruy-Filho L Wieck A Bauer ME. Evidence for immune activation and resistance to glucocorticoids following childhood maltreatment in adolescents without psychopathology. Neuropsychopharmacology (2017) 42:2272–82. 10.1038/npp.2017.137 28664925 PMC5603807

[67] Desantis SM Baker NL Back SE Spratt E Ciolino JD Moran-Santa Maria M. Gender differences in the effect of early life trauma on hypothalamic-pituitary-adrenal axis functioning. Depress Anxiety (2011) 28:383–92. 10.1002/da.20795 21328636 PMC3243643

[68] Heim C Newport D Heit S Graham YP Wilcox M Bonsall R. Pituitary-adrenal and autonomic responses to stress in women after sexual and physical abuse in childhood. JAMA (2000) 284:592–7. 10.1001/jama.284.5.592 10918705

[69] Heim C Mletzko T Purselle D Musselman DL Nemeroff CB. The dexamethasone/corticotropin-releasing factor test in men with major depression: role of childhood trauma. Biol Psychiatry (2008) 63:398–405. 10.1016/j.biopsych.2007.07.002 17825799

[70] Kim K Mennen FE Trickett PK. Patterns and correlates of co-occurrence among multiple types of child maltreatment. Child Fam Soc Work (2017) 22:492–502. 10.1111/cfs.12268 29225485 PMC5720384

[71] Pechtel P Lyons-Ruth K Anderson CM Teicher MH. Sensitive periods of amygdala development: the role of maltreatment in preadolescence. Neuroimage (2014) 97:236–44. 10.1016/j.neuroimage.2014.04.025 24736182 PMC4258391

[72] Putnam FW. Ten-year research update review: child sexual abuse. J Am Acad Child Adolesc Psychiatry (2003) 42:269–78. 10.1097/00004583-200303000-00006 12595779

[73] Hagger-Johnson G Mõttus R Craig LCA Starr JM Deary IJ. Pathways from childhood intelligence and socioeconomic status to late-life cardiovascular disease risk. Heal Psychol. (2012) 31:403–12. 10.1037/a0026775 22309883

[74] Noll JG Zeller MH Trickett PK Putnam FW. Obesity risk for female victims of childhood sexual abuse: a prospective study. Pediatrics (2007) 120:e61–7. 10.1542/peds.2006-3058 17606550

[75] Gluckman PD Hanson MA Beedle AS. Early life events and their consequences for later disease: a life history and evolutionary perspective. Am J Hum Biol. (2007) 19:1–19. 10.1002/ajhb.20590 17160980

[76] Blanco L Nydegger LA Camarillo G Trinidad DR Schramm E Ames SL. Neurological changes in brain structure and functions among individuals with a history of childhood sexual abuse: a review. Neurosci Biobehav Rev. (2015) 57:63–9. 10.1016/j.neubiorev.2015.07.013 26363666

[77] Villano CL Cleland C Rosenblum A Fong C Nuttbrock L Marthol M. Psychometric utility of the childhood trauma questionnaire with female street-based sex workers. J Trauma Dissociation (2004) 5:33–54. 10.1300/J229v05n04_03 16957783 PMC1560176

[78] Okuda PMM Klaiman C Bradshaw J Reid M Cogo-Moreira H. Assessing risk of bias in randomized controlled trials for autism spectrum disorder. Front Psychiatry (2017) 8:265. 10.3389/fpsyt.2017.00265 29238311 PMC5712530

